# An Experimental Investigation of the Relationship Between Emotion Regulation Flexibility, Negative Affect and Posttraumatic Stress Disorder

**DOI:** 10.1007/s10608-024-10536-3

**Published:** 2024-10-04

**Authors:** Madeleine Lim, Angela Nickerson, Philippa Specker

**Affiliations:** School of Psychology, University of New South Wales, Sydney, NSW 2052 Australia

**Keywords:** Emotion regulation, Flexibility, PTSD, Reappraisal, Distraction, Experimental

## Abstract

**Purpose:**

Emerging research investigating mechanisms underpinning PTSD has identified emotion regulation (ER) flexibility – the ability to flexibly use ER strategies according to contextual demands – as one promising mechanism. To date, however, no study has investigated whether brief training in ER flexibility can minimise negative affect elicited from evocative stimuli. This study investigated the impact of instructed ER flexibility on emotional responding in probable PTSD.

**Methods:**

Participants (*N =* 148) viewed images that differed in negative emotional intensity (high or low). For each image, participants followed instructions to adopt either a flexible or inflexible ER approach through randomisation to either an *ER flexible* condition or one of three control conditions (*Inflexible Reappraisal*,* Inflexible Distraction*,* Context Insensitive*). In the *ER Flexible* condition, participants were instructed to switch between distraction and reappraisal according to the emotional intensity of the image. The control conditions required participants to either employ a single ER strategy (*Inflexible Distraction* and *Inflexible Reappraisal*) or switch between strategies in a way that did not align with image intensity (*Context Insensitive*). Negative affect was rated after each image.

**Results:**

Participants with probable PTSD in the *ER Flexible* condition demonstrated the lowest levels of negative affect. For participants without probable PTSD, negative affect did not differ between the ER conditions.

**Conclusions:**

Findings suggest that individuals with probable PTSD benefitted from being instructed in ER flexibility. This finding supports the adaptiveness of ER flexibility and provides a preliminary temporal link between instructed ER flexibility and improved emotional responding for those with PTSD.

**Supplementary Information:**

The online version contains supplementary material available at 10.1007/s10608-024-10536-3.

Posttraumatic stress disorder (PTSD) is a debilitating psychological illness characterized by significant emotion dysregulation (American Psychiatric Association, 2013). To further refine our clinical interventions, we must develop a more precise understanding of the mechanisms underpinning PTSD. One promising candidate mechanism underlying the relationship between trauma exposure and PTSD is emotion regulation (ER) flexibility (Levy-Gigi et al., 2016). ER flexibility refers to the ability to select and switch between different ER strategies according to dynamic contextual demands (Aldao et al., 2015). ER flexibility is conceptually aligned with existing theoretical frameworks, including psychological flexibility (Kashdan & Rottenberg, 2010) and coping flexibility (Cheng, 2001), which emphasise the adaptive benefit of varying one’s use of regulatory strategies. However, ER flexibility is distinct in its explicit emphasis on the centrality of aligning one’s ER behaviour according to the specific contextual demands of each situation. In this way, ER flexibility has been defined as a multi-componential process encompassing one’s sensitivity to situational context (*context sensitivity*), one’s repertoire of ER strategies (*strategy repertoire)*, and one’s ability to monitor strategy efficacy (*feedback responsiveness*) (Chen & Bonanno, 2021). The synchrony of these three components underpins ER flexibility and drives adaptive psychological outcomes (Chen & Bonanno, 2021).

Given the complexity of ER flexibility as a multi-componential process, measuring ER flexibility in a way that accounts for the dynamic interplay between strategy use and situational context has been a significant challenge. While there is no consensus on the best way to measure ER flexibility, popular methodological approaches have included survey-based questionnaires [e.g., Context Sensitive Index (Chen & Bonanno, 2021); Flexible Regulation of Emotional Expression Scale (Burton & Bonanno, 2016)], ecological momentary assessment or experience sampling methodology (e.g., Chen et al., 2024; English et al., 2020), and experimental paradigms (e.g., Sheppes et al., 2011; 2014). Of these, the current study will focus on experimental methodology. This provides a first step to understanding causal associations between mechanisms and outcomes. One of the dominant experimental paradigms used to measure ER flexibility is the regulatory selection paradigm (Sheppes et al., 2011). This experimental paradigm is informed by Aldao and colleagues’ (2015) conceptualisation of ER flexibility, which focuses on the aforementioned components of *context sensitivity* and *strategy repertoire*. In this paradigm, participants are instructed to minimise their distress elicited by changing contextual demands - low and high-intensity negative stimuli (e.g., negative images, electric shocks, or distressing phrases) - by selecting from an available strategy repertoire of either reappraisal^1^ or distraction^2^. In line with empirical evidence, ER flexibility is operationalised in this paradigm as the preferential selection of reappraisal when confronted with stimuli low in emotional intensity, and distraction for stimuli high in emotional intensity (Fine et al., 2021; Levy-Gigi et al., 2016; Sheppes et al., 2011). This operationalisation rests on prior experimental research consistently demonstrating that healthy individuals spontaneously switch their ER strategy based on changes in the emotional intensity of stimuli. Specifically, individuals overwhelmingly choose to distract in high emotional intensity contexts and reappraise in low emotional intensity contexts (Fine et al., 2021; Levy-Gigi et al., 2016; Specker et al., 2023). This pattern of regulatory selection has been corroborated theoretically and using neurophysiological data (Sheppes, 2020). Distraction is thought to be suitable for managing emotions in high-intensity contexts as it allows individuals to redirect their attention away from the triggering stimulus (Sheppes & Meiran, 2008). In doing so, distraction prevents premature emotional processing and the subsequent development of disproportionately negative emotion (Sheppes, 2020). Using neuroimaging data, distraction is associated with earlier and stronger modulation of the Late Positive Potential (LPP; an electrocortical indicator of effective processing of emotionally arousing information). Strong, early modulation of LPP results in greater activation of the neural network suppressing amygdala activity associated with fear, making distraction more suited to high-intensity situations (Kanske et al., 2011; McRae et al., 2010; Sheppes et al., 2011). Conversely, individuals exhibit lower negative emotionality and emotional reactivity in low-intensity situations (Sheppes, 2020). Therefore, reappraisal may be more suitable for low-intensity situations where individuals can successfully engage and accurately reinterpret the meaning of the emotional stimuli to downregulate their emotional response (Sheppes, 2020). In EEG studies, reappraisal has been shown to allow for deeper, more meaningful processing of emotional stimuli. This is evidenced by late modulation of the LPP, and was associated with lower activation of the neural network governing fear-based responses to emotional situations via amygdala activity (Kanske et al., 2011; McRae et al., 2010; Sheppes et al., 2011). Thus, the tendency to reappraise low intensity situations and distract from high intensity situations is considered the most adaptive pattern of ER flexibility.

Burgeoning experimental evidence demonstrates a strong association between ER flexibility and PTSD using the regulatory selection paradigm (Fine et al., 2021; Levy-Gigi et al., 2016). Among university students and child sexual assault survivors, those with higher levels of PTSD exhibited lower levels of ER flexibility compared to matched controls (Fine et al., 2021). Further, Levy-Gigi and colleagues (2016) found that ER flexibility moderated the relationship between PTSD and trauma exposure whereby greater trauma exposure only predicted PTSD if war-exposed firefighters exhibited lower levels of ER flexibility. Importantly, this well-established dose-response relationship between trauma exposure and PTSD was non-existent for firefighters with high regulatory flexibility (Levy-Gigi et al., 2016). These studies present two critical findings: first, that deficits in ER flexibility are associated with more severe PTSD symptomatology, and second, that ER flexibility may be protective against the development of PTSD following trauma. Although these findings advance our current understanding of the mechanisms underpinning PTSD development, the conclusions drawn from these paradigms remain correlational in nature. Instead, an experimental paradigm that manipulates, rather than measures, levels of ER flexibility would allow us to test the mechanistic influence of ER flexibility in PTSD. One such paradigm is the instructed ER flexibility paradigm (Specker & Nickerson, 2023).

In line with the prevailing conceptualization of ER flexibility (Aldao et al., 2015), Specker and Nickerson’s instructed paradigm (2023) operationalises ER flexibility as the possession of (1) a repertoire of ER strategies and, (2) context sensitive strategy use. Here, the ER strategy repertoire comprises both reappraisal and distraction, and context sensitivity is operationalised as the matching of ER strategies in line with changing contextual demands (changes in image intensity). Unlike Sheppes’ (2011) regulatory selection paradigm which measures the natural regulatory tendencies of participants (*spontaneous ER*), the instructed ER flexibility paradigm experimentally *manipulates* ER flexibility. To do this, participants are instructed to adopt either a flexible or inflexible ER approach when confronted with negative images. Emotional responses are elicited via negative images equally distributed across low and high emotional intensity. This creates two distinct emotional contexts; high emotional intensity stimuli and low emotional intensity stimuli. The paradigm comprises four conditions. To manipulate ER flexibility, participants in the *ER Flexible* condition are instructed to distract during high-intensity emotional stimuli and reappraise low-intensity emotional stimuli. The *ER Flexible* condition represents a wide ER strategy repertoire and high context sensitivity, in line with the prevailing conceptualization of ER flexibility (Bonanno & Burton, 2013). To create a fully crossed design, three additional control conditions are included: *Context Insensitive*,* Inflexible Distraction*,* Inflexible Reappraisal*. In the first control condition, *Context Insensitive*, participants are instructed to adopt the inverse pattern of the *ER Flexible* condition. That is, participants reappraise high-intensity emotional stimuli and distract during low-intensity emotional stimuli. This condition functions as a control to test whether the benefit of a flexible ER approach on emotional responding is driven by both a wide strategy repertoire and high context sensitivity (*ER Flexible*) or simply just a wide strategy repertoire without any context sensitivity (*Context Insensitive*). Two additional inflexible control conditions are included. In these conditions, participants are instructed to either exclusively reappraise (*Inflexible Reappraisal*) or distract (*Inflexible Distraction*) regardless of changing contextual demands. Here, participants are instructed to adopt a narrow ER strategy repertoire and poor context sensitivity. Following each image, emotional responding is indexed via self-reported negative affect. Previous research using this paradigm found that individuals with high anxiety reported lower negative affect when instructed to adopt an ER flexible approach (Specker & Nickerson, 2023).

There is mounting evidence to suggest that lower levels of ER flexibility are implicated in PTSD (Fine et al., 2021; Levy-Gigi et al., 2016). However, there is an apparent deficit of evidence causally relating ER flexibility to emotional responding in PTSD. Considering the remaining gaps in the PTSD literature, the present study used the instructed ER flexibility paradigm to investigate the mechanistic influence of ER flexibility on emotional responding among trauma-exposed individuals with and without probable PTSD. In line with Specker and Nickerson’s (2023) findings demonstrating a temporal link between ER flexibility and anxiety, we predicted that being guided in how to adopt an ER flexible approach when confronted with trauma-salient stimuli would be most effective at downregulating negative affect for those with probable PTSD. This is because PTSD, like anxiety, has been associated with marked deficits in ER flexibility relative to cohorts without PTSD (Fine et al., 2021). Specifically, we hypothesised that participants with probable PTSD in the *ER Flexible* condition would exhibit significantly lower negative affect in response to trauma-salient stimuli than those in the inflexible ER control conditions. For those without probable PTSD, we did not expect to detect a pronounced difference in emotional responding between the instructed conditions.

## Materials and Methods

### Participants

**Fig. 1:**
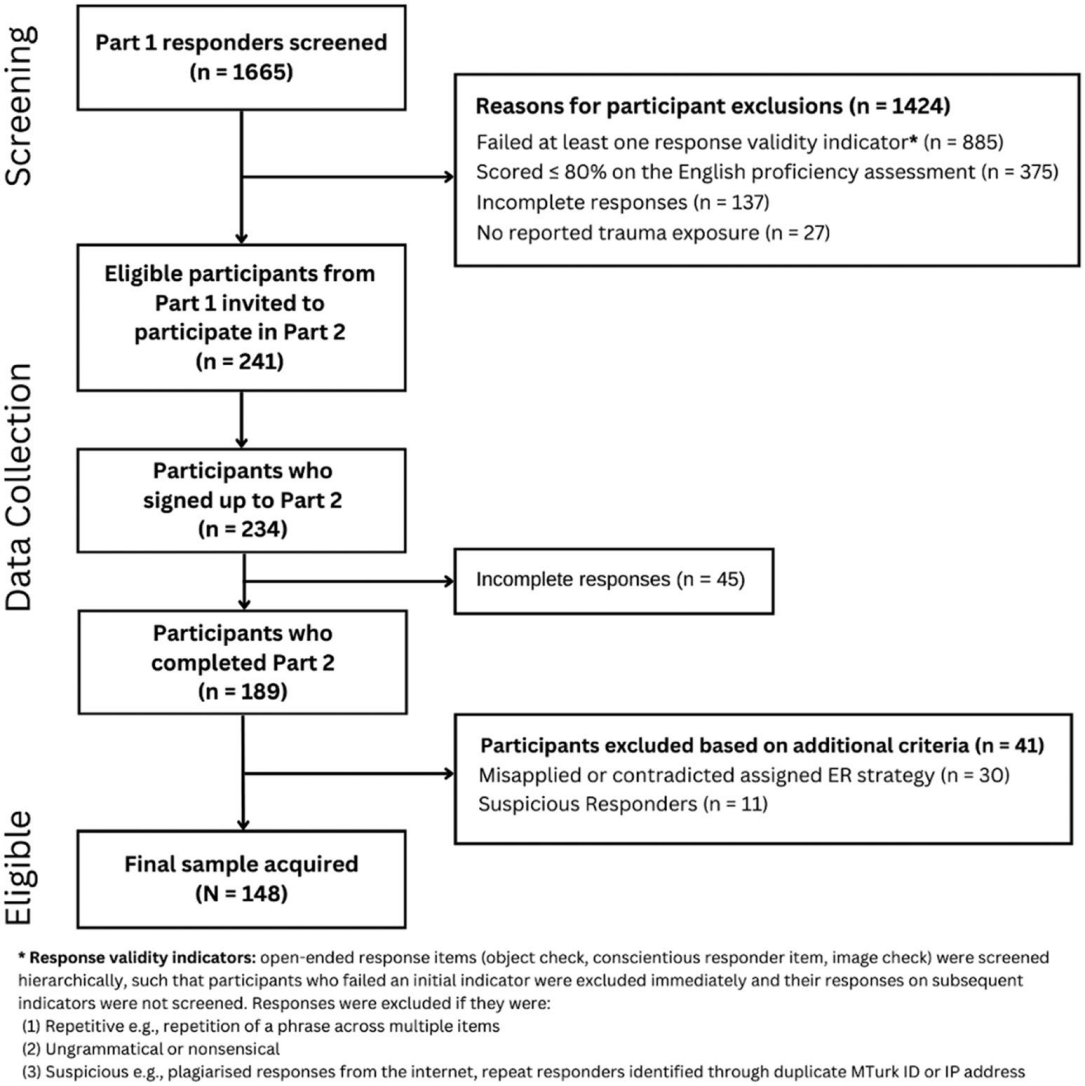
Flowchart depicting participant exclusions during the recruitment and screening phases of Part 1 and 2

Participants were recruited via Amazon’s Mechanical Turk (MTurk) across June and July 2023. MTurk is the most widely used platform for online studies as it is low-cost and yields high-quality data (Aguinis et al., 2021). Additionally, MTurk yields similar prevalence rates of trauma exposure and psychopathology as lab-based samples (van Stolk-Cooke et al., 2018). Eligibility criteria required participants to have experienced a Criterion A traumatic event according to the Fifth Edition of the Diagnostic Statistical Manual for Mental Disorders (American Psychiatric Association, 2013), be fluent in English, be at least eighteen years old, reside in the United States, and have completed at least 1000 Human Intelligence Tasks (HIT) with a HIT approval ratio (HAR) of at least 99%. Several response validity indicators were incorporated into a Part 1 screener in line with established recommendations to identify and exclude suspicious responders (e.g., bots) and inattentive responders (See Supplement A of the Supplemental Materials). The recruitment and screening process for parts 1 and 2 are depicted in Fig. 1. An a priori power analysis conducted via GPower 3.1.9.7. was conducted to determine our target sample size. This indicated that a sample size of 126 participants was required to detect a small to medium effect size (*f* = 0.03) with 80% power (1-b) and an error rate (a) of 0.05. The final sample comprised 148 participants.

### Measures

#### Demographics

Participants provided their age, gender, ethnic background, and level of educational attainment.

#### Trauma Exposure

The Life Events Checklist – Fifth Edition (LEC-5) (Weathers et al., 2013) was used to screen for lifetime exposure to potentially traumatic events. The LEC-5 comprises 16 potentially traumatic events according to the DSM-5 Criterion A definition of a traumatic event. For each event, participants select any of the following response options that apply: (1) Happened to me, (2) Witnessed it, (3) Learned about it, (4) Part of my job, (5) Not sure, or (6) Doesn’t apply (Weathers et al., 2013). An item was considered endorsed if either (1), (2) or (4) were selected. A total score was then computed by summing endorsed items. To reduce demand characteristics during the initial screening phase, 20 ‘filler’ items describing neutral or positive events were added to the existing items (Jobson et al., 2022). The LEC-5 has good convergent validity with the gold-standard Clinician-Administered PTSD Scale (Gray et al., 2004) and is routinely used in trauma-exposed US samples (Gray et al., 2004).

#### PTSD Symptoms

The Posttraumatic Stress Disorder Checklist for DSM-5 (PCL-5) (Weathers et al., 2013) indexed PTSD symptom severity. The PCL-5 is a 20-item measure that assesses DSM-5 PTSD symptoms (Weathers et al., 2013). Each item is measured on a 5-point Likert scale (0 = *Not at all*, 4 = *Extremely*). Items are added to generate a total score ranging from 0 to 80 (van Stolk-Cooke et al., 2018). The PCL-5 provides a clinical cut-off score, with scores above 33 suggesting a probable PTSD diagnosis (Verhey et al., 2018). The PCL-5 is a gold-standard tool for assessing PTSD symptoms, with good construct and convergent validity with other measures of PTSD (McDonald & Calhoun, 2010). The PCL-5 demonstrated excellent internal consistency in the current sample (α = 0.964).

#### Emotional Stimuli

Images presented in this study were identical to those used in Sheppes and colleagues’ (2011) regulatory selection paradigm. This set comprises 30 trauma-related International Affective Picture System (IAPS; Bradley & Lang, 2017) images depicting interpersonal violence, deceased people and animals, civil unrest, mutilated bodies, and insects. There were 15 low emotional intensity images (Mean Valence_Low_ = 3.41; Mean Arousal_Low_ = 5.01) and 15 high emotional intensity images (Mean Valence_Low_ = 1.99; Mean Arousal_Low_ = 6.12), categorised based on their normative ratings for arousal (Sheppes et al., 2011). To improve validity, image content was approximately matched across low- and high-emotional intensity categories (Levy-Gigi et al., 2016).

#### Nature Images

A total of nine images depicting a natural landscape were used in the present study to improve executive attention by minimising participant fatigue (Gamble et al., 2014). A nature image was presented after every set of three negative images. Images depicted scenes of waterfalls, lakes, and beaches. All images were approximately matched in content.

#### Negative Affect

Consistent with past ER experimental paradigms (Sheppes et al., 2014; Specker & Nickerson, 2023), negative affect was measured using a single self-report item following the presentation of each negative image. Participants were asked to rate how negatively they felt after viewing the experimental image and using their assigned ER strategy (“*How negative did the picture make you feel?*”) on a 9-point Likert scale (1 = *Not negative at all*, 9 = *Very negative)* (Sheppes et al., 2014). A mean score was calculated to index overall negative affect.

#### Manipulation Check

Consistent with past ER experimental paradigms, participants were asked to, “*Briefly describe what you thought about while viewing the image you just saw*” to verify the correct implementation of their assigned ER strategy (Sheppes et al., 2011; Specker & Nickerson, 2023). A total of 16 open-ended validity checks were included in the study. Validity checks were presented after each of the eight training and practice items and validity checks were also presented an additional eight times randomly throughout the experimental phase. Consistent with previous studies (e.g., Fine et al., 2021; Levy-Gigi et al., 2016; Specker & Nickerson, 2023), responses were inspected by a single rater blinded to the participants’ instructed ER condition allocation. Responses were flagged if they contradicted the experimental instructions, for example, if a participant distracted despite being told to reappraise, or demonstrated a gross misapplication of the assigned strategy, for example, not distracting or reappraising. Participants who failed four or more checks were excluded from the analysis (see Fig. 1). Consistent with similar previous studies, 22% of participants were excluded for failing this manipulation check (Specker & Nickerson, 2023).

### Procedure

The current study was conducted online using Qualtrics. Data collection occurred over two sessions – Part 1 and 2 – conducted approximately seven days apart. Following informed consent, participants completed the English proficiency test, demographic questionnaire and the attention and validity indicator items (object check, conscientious responder check, and image check; see Supplement A of the Supplemental Materials). Participants then completed a modified version of the LEC-5 containing additional neutral and positive events to assess levels of trauma exposure while reducing demand characteristics. After Part 1, participants were debriefed and received USD$1.50 in compensation.

Eligible participants were sent an advertisement and survey link for Part 2 via MTurk. After consenting to participate in Part 2, trauma exposure was re-assessed, this time using only the original LEC-5 (i.e., with no filler items), and PTSD symptom severity was measured using the PCL-5.

Next, participants proceeded to the instructed ER flexibility paradigm. The current study replicated the experimental procedure for the instructed ER flexibility paradigm outlined by Specker and Nickerson (2023), with the addition of nature images between experimental blocks. Nature images were added to minimise fatigue by improving executive attention (Gamble et al., 2014). The instructed ER flexibility paradigm comprised three phases: teaching, practice, and experimental.

In the teaching phase, participants were briefly introduced to the experimental task and oriented to the negative affect scale. Participants then received instructions on distraction and reappraisal via written text and instructional videos (links to the videos and instructions can be found in Supplement B of the Supplemental Materials). The duration of each instructional video was 2 min and 30 s. The videos provided a brief explanation of the strategy and a demonstration of how to use the strategy whilst viewing a negative image like those shown in the experimental phase. After viewing each video, participants practised implementing the target strategy while viewing one low-intensity image and one high-intensity image. The order in which participants learnt the two ER strategies was counterbalanced.

In the practice phase, participants were provided further details on the experimental procedure and reminded to only use the assigned strategy. Then, participants completed two distraction trials and two reappraisal trials, each comprising a low and high-intensity image. In each practice trial, participants first previewed the image for one second. Then, participants were informed in bold written text which strategy to employ when the image returned. When the participant had finished reading the instruction, the same image reappeared for six seconds whilst the participant attended to the image and implemented their instructed strategy. Each participant was asked to complete an open response item asking them to describe how they distracted or reappraised the image. These responses were blindly coded after completion. Participants then rated their negative affect.

**Fig. 2:**
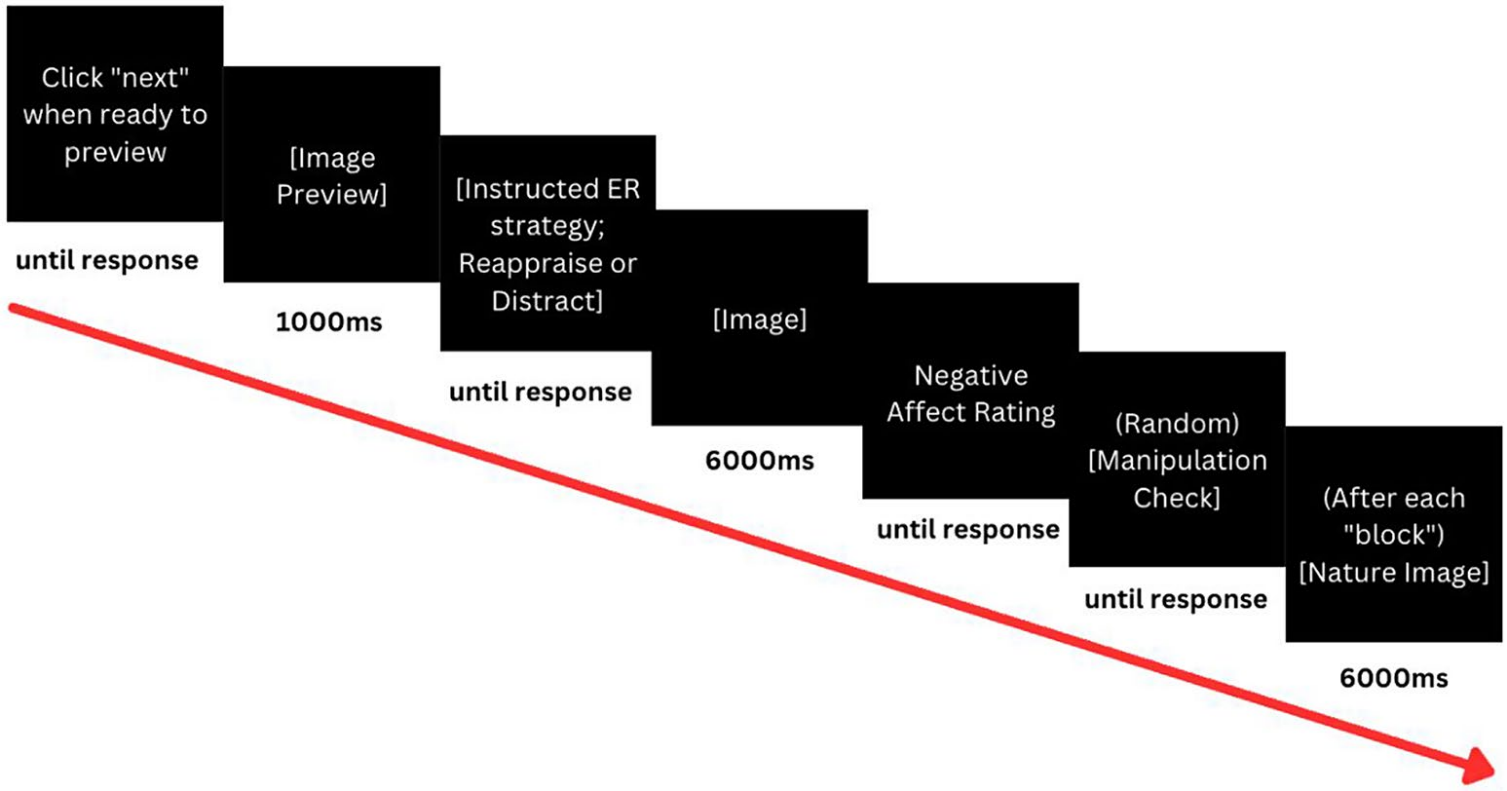
Illustration of the instructed ER flexibility experimental trial structure. Ms = milliseconds

In the experimental phase, participants were randomised to one of four conditions: *ER Flexible*,* Context Insensitive*,* Inflexible Distraction*, and *Inflexible Reappraisal.* Participants assigned to the *ER Flexible* condition were instructed to use distraction during high-intensity images and reappraisal during the presentation of low-intensity images. Participants assigned to the *Context Insensitive* condition were instructed to use reappraisal during high-intensity images and distraction during low-intensity images (i.e., the inverse pattern to the *ER Flexible* condition). In *Inflexible Distraction* and *Inflexible Reappraisal* conditions, participants were instructed to employ a single ER strategy (either reappraisal or distraction) regardless of changes in contextual demands (image intensity). Participants were not informed that the experimental images varied in negative emotional intensity. Images were arranged into ten “blocks”. Each “block” contained three negative images of the same emotional intensity, so participants used the same ER strategy when attending to images in the same “block”. Images were arranged this way to minimise the potential added cognitive load posed by being involved in the experimental conditions that required participants to switch between the two strategies (*ER Flexible* and *Context Insensitive*) (Specker & Nickerson, 2023). The order of the manipulation checks, images, and blocks was randomised and then fixed across conditions, in line with Specker and Nickerson’s (2023) experimental procedure. The structure and timing of the experimental trials are shown in Fig. 2. Part 2 took approximately 40 min to complete. After the experiment, participants were debriefed and compensated with USD$4.50. The [De-Identified] Human Research Ethics Committee provided ethics approval ([De-identified]).

### Data Analysis

Statistical analyses were conducted using IBM SPSS version 29. Chi-square tests, a one-way Analysis of Variance (ANOVA), and an independent samples t-test were conducted to identify between-group differences (i.e., between the probable PTSD groups and between the instructed ER conditions) in demographic characteristics. A paired samples t-test was used to verify that the experimental manipulation of image intensity to generate distinct ‘low’ and ‘high’ negative emotional intensity contexts was successful. For the main analysis, a 2 (probable PTSD) x 4 (instructed ER conditions) Analysis of Covariance (ANCOVA) with planned comparisons was used to examine whether instructing participants in ER flexibility (*ER Flexible* condition) would result in lower negative affect compared to the three control conditions (*Context Insensitive*,* Inflexible Distraction*,* Inflexible Reappraisal*), particularly among those with probable PTSD. Instructed ER conditions (*ER Flexible*,* Context Insensitive*,* Inflexible Distraction*,* Inflexible Reappraisal*) and probable PTSD groups (*probable PTSD*,* no PTSD*) were entered as between-subject factors. Since age differed between the instructed ER conditions, it was entered into the model as a covariate. The Benjamini-Hochberg false discovery rate procedure was used to statistically control for the use of multiple comparisons (Benjamini & Hochberg, 1995). This procedure is less stringent than the Bonferroni correction, and considered a valid alternative that balances both the objectives of reducing the family wise error rate while maximising statistical power to detect a true effect (Ioannidis, 2005; Perneger, 1998).

## Results

### Sample Characteristics and Probable PTSD

Sociodemographic characteristics are provided in Table 1. Participants were predominantly male (54.6%) and predominantly white (78.4%). 24 (16.2%) participants met the criteria for a probable PTSD diagnosis on the PCL-5, while 124 (83.8%) participants did not met criteria for probable PTSD (Ashbaugh et al., 2016). Frequencies of participant exposure to potentially traumatic events can be found in Supplement C of the Supplemental Materials. The most frequently endorsed traumatic events were transportation accidents (77%), natural disasters (56.8%), physical assault (48.6%), and a life-threatening illness or injury (43.2%).

**Table 1:** Demographic characteristics for the overall sample

Demographic Variables	M (SD) or *n* (%)
Age	42.2 (11.4)
Gender	81 (54.6%) male, 64 (43.2%) female
Other	1 (0.7%)
Prefer not to answer	2 (1.4%)
Cultural background	
White or Caucasian	116 (78.4%)
Black or African American	12 (8.1%)
Latino or Hispanic	5 (3.4%)
American Indian or Alaska native	1 (0.7%)
Asian or South Asian	6 (4.1%)
Native Hawaiian or Pacific Islander	1 (0.7%)
Other	4 (2.7%)
Prefer not to answer	3 (2.0%)
Employment status	
Employed full-time	110 (74.3%)
Employed part-time	13 (8.8%)
Employed casually	4 (2.7%)
Unemployed	4 (2.7%)
Homemaker	3 (2.0%)
Retired	11 (7.4%)
Highest educational attainment	
Junior High	1 (0.7%)
Senior High	16 (10.8%)
Some college credit	31 (20.9%)
Undergraduate Degree	75 (50.7%)
Vocational Qualification	4 (2.7%)
Masters Degree	17 (11.5%)
Doctoral Degree	2 (1.4%)
Prefer not to answer	2 (1.4%)

*Note*: *N* = 148

### Preliminary Analyses

To verify random allocation and test whether there were any systematic differences between instructed ER conditions or probable PTSD groups, the conditions and groups were compared across demographic variables.

#### Instructed ER Conditions

Chi-squared tests indicated no significant differences between instructed ER conditions in gender, *χ*^*2*^ (3, *N* = 145) = 7.47, *p* = .058, *ϕ* = 0.04, ethnicity, *χ*^*2*^ (3, *N* = 148) = 0.473, *p* = .925, *ϕ* = 0.05, employment, *χ*^*2*^ (3, *N* = 148) = 0.62, *p* = .891, *ϕ* = 0.04, or education, *χ*^*2*^ (3, *N* = 146) = 3.06, *p* = .383, *ϕ* = 0.07. However, a one-way ANOVA showed significant differences between the instructed ER conditions with respect to age, *F*(3, 144) = 2.70, *p* = .048, η_p_^2^ = 0.05. Thus, age was controlled for in the main analyses. Overall, these findings show that random allocation was largely successful.

#### Probable PTSD Groups

Chi-square tests indicated that probable and non-probable PTSD groups did not significantly differ in ethnicity, *χ*^*2*^ (31, *N* = 148) = 1.22, *p* = .270, *ϕ* = 0.09, employment, *χ*^*2*^ (1, *N* = 148) = 2.36, *p* = .124, *ϕ* = 0.13, education, *χ*^*2*^ (1, *N* = 146) = 0.81 *p* = .369, *ϕ* = 0.07, or gender, *χ*^*2*^ (3, *N* = 148) = 6.61, *p* = .085, *ϕ* = 0.21. An independent-samples t-test showed that age did not significantly differ among those with and without probable PTSD, *t*(148) = 0.35, *p* = .073, *d* = 0.08. Further, the distribution of participants in the probable PTSD vs. non-probable PTSD groups did not significantly differ between the instructed ER conditions, *χ*^*2*^ (3, *N* = 148) = 4.90, *p* = .179, *ϕ* = 0.12. These findings demonstrate that there were no systematic differences between the probable and non-probable PTSD groups.

#### Verification of Image Intensity

A paired samples t-test indicated that there was a significant difference between mean negative affect for images of low (*M* = 2.56, *SD* = 1.25) and high (*M* = 5.30, *SD* = 1.83) negative emotional intensity, *t*(145) = 24.92, *p* < .001. This shows that the low- and high-intensity images elicited two distinct levels of negative emotional responses in line with their pre-determined intensity, thus indicating that the experimental manipulation of emotional context was valid.

### ANCOVA Analyses

A 2 (probable PTSD Group) x 4 (Instructed ER Condition) between-subjects ANCOVA with planned comparisons was performed to test the hypotheses of the current study. For completeness, the results of the overall ANCOVA model are presented in Table 2 and all pairwise comparisons are presented in Supplement D of the Supplemental Materials. As expected, there was a significant main effect for probable PTSD group, *F*(1, 138) = 10.80, *p* = .001, η_p_^2^ = 0.05, such that participants with probable PTSD (*M* = 4.93, *SD* = 0.32) reported significantly greater negative affect than those without probable PTSD (*M* = 3.80, *SD* = 0.12). The main effect of instructed ER condition on negative affect was not significant, *F*(3, 138) = 2.23, *p* = .087, η_p_^2^ = 0.05. The interaction effect between instructed ER condition and probable PTSD groups was significant, *F*(3, 138) = 6.46, *p* = .016, η_p_^2^ = 0.05. This indicated that the impact of the instructed ER conditions on participant negative affect differed according to the participant’s probable PTSD status. Planned comparisons exploring the impact of the instructed ER conditions on negative affect among the probable and non-probable PTSD groups were separately conducted to disaggregate this effect.

**Table 2:** ANCOVA of the effect of instructed ER and PTSD on negative affect

Variables	F	*p*	η_*p*_^2^
Covariate			
Age	*F*(1,138) = 0.58	0.447	0.073
Main Effect			
Probable PTSD group	*F*(1,138) = 10.80	0.001	0.073
Instructed ER condition	*F*(3,138) = 2.23	0.087	0.046
Interaction			
PTSD x Condition	*F*(3,138) = 3.58	0.016	0.072

Note. *N* = 148. R Squared = 0.158 (Adjusted R Squares = 0.101). Probable PTSD diagnosis indicated by scoring 33 or more on the PTSD Checklist for DSM-5 (PCL-5)

### Planned Comparisons

**Fig. 3:**
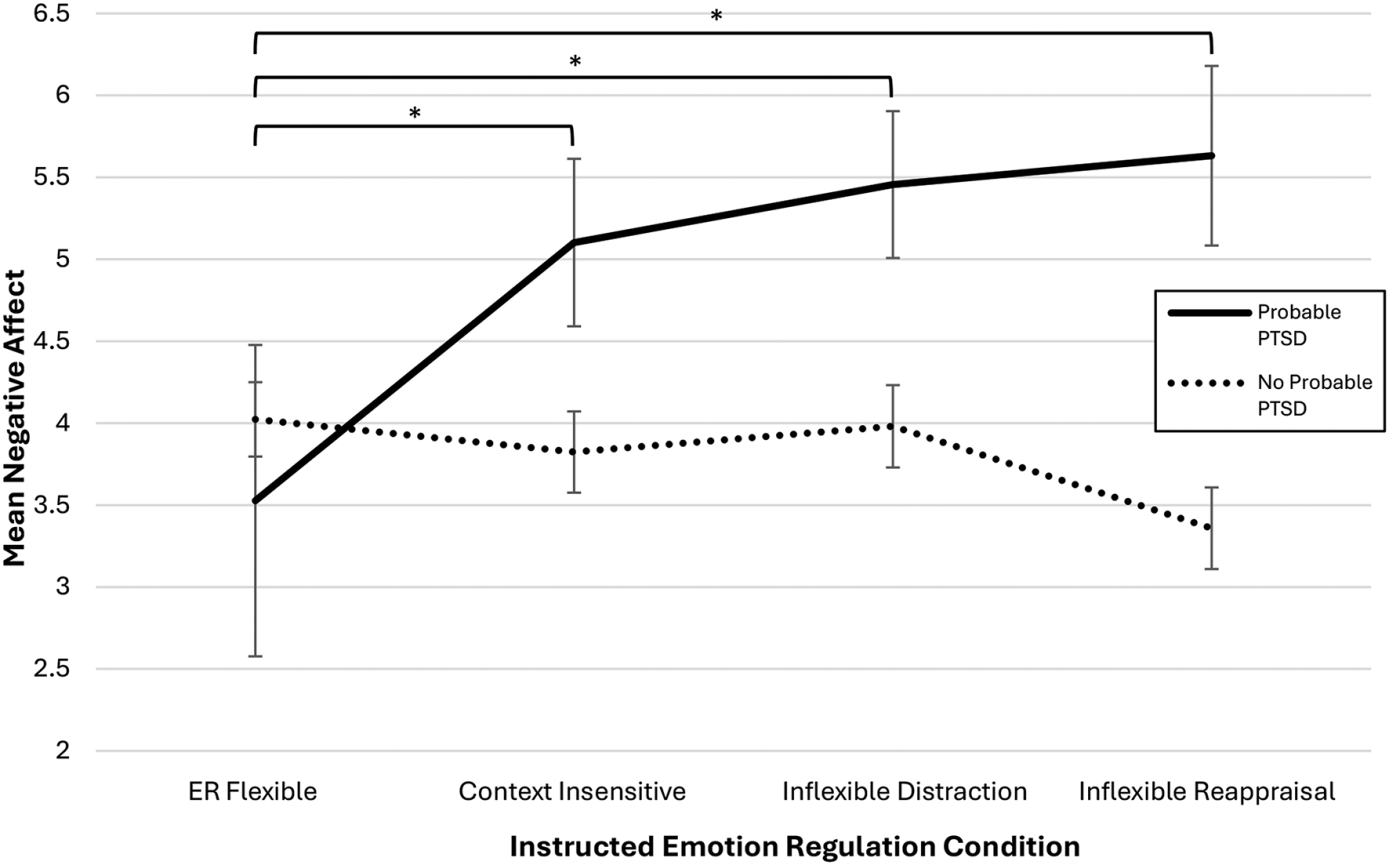
Mean negative affect among probable PTSD groups across instructed ER conditions. *Note.* Error bars represent ± 1 standard error. Significance brackets show differences between conditions among participants with probable PTSD. The significance brackets show statistically significant differences between the *ER Flexible* condition and the *Context Insensitive* (*p =* .028), *Inflexible Distraction* (*p =* .005), and *Inflexible Reappraisal* (*p =* .047) conditions

Testing whether instruction in ER flexibility (ER Flexible condition) results in lower negative affect for those with probable PTSD. For participants with probable PTSD, mean negative affect significantly differed between the instructed ER conditions, *F*(3, 138) = 3.50, *p* = .017, η_p_^2^ = 0.07. Specifically, planned comparisons indicated that, among participants with probable PTSD, those in the *ER Flexible* condition (*M* = 3.53, *SE* = 0.45) exhibited significantly lower mean negative affect when compared to those in the *Inflexible Distraction* (*M =* 5.46, *SE* = 0.51), *p* = .005, 95% *CI* [-3.28, -0.58], *Context Insensitive* (*M* = 5.10, *SE* = 0.55), *p* = .028, 95% *CI* [-2.97, -0.18], and *Inflexible Reappraisal* (*M* = 5.63, *SE* = 0.95) conditions, *p* = .047, 95% *CI* [-4.18, -0.03]. These comparisons remained significant after evaluating them against corrected alpha significance levels, calculated using the Benjamini-Hochberg procedure (adjusted α values were: α = 0.0167 [comparison 1], α = 0.0333 [comparison 2], α = 0.05 [comparison 3]). Mean negative affect did not significantly differ among the instructed ER conditions for participants without probable PTSD, *F*(3, 138) = 1.69, *p* = .171, η_p_^2^ = 0.04. The mean negative affect for those with and without probable PTSD in each instructed ER condition is depicted in Fig. 3.

## Discussion

The current study used an instructed ER flexibility paradigm to assess whether ER flexibility functioned as a mechanism influencing emotional responding in those with probable PTSD. Our results revealed that training trauma-exposed participants with probable PTSD in ER flexibility was the most effective way to manage negative affect relative to all other regulatory approaches tested. This supported our hypothesis, which posited that individuals with probable PTSD would derive the greatest benefit from being instructed in ER flexibility. It is also noteworthy that the same pattern of findings was not found for those without probable PTSD. Although the present study was preliminary, our findings may indicate the potential protective effects of ER flexibility for those with PTSD. The present findings thus build upon the existing literature. To date, the relationship between PTSD and ER flexibility has only been demonstrated in a correlational manner (Fine et al., 2021; Levy-Gigi et al., 2016). Studies have shown associations between greater ER flexibility deficits and greater severity of PTSD symptomatology (O’Brien et al., 2023). Such ER flexibility deficits include limited access to ER strategies, poor emotional awareness, and an inability to select appropriate strategies to manage emotions (O’Brien et al., 2023; Spikol et al., 2024). However, an integral requirement for classifying ER flexibility as a psychological mechanism underpinning PTSD is to demonstrate this relationship causally (Ehring et al., 2022). Specifically, high levels of ER flexibility must be shown to induce improvements in subsequent emotional responding. To our knowledge, this is the first study to experimentally manipulate ER flexibility and quantify its real-time impact on emotional responding in an analogue probable PTSD sample.

Our findings accord with prior research by Levy-Gigi and colleagues (2016), which provided indicators of the mechanistic influence of ER flexibility on the development of PTSD. Levy-Gigi and colleagues found that the relationship between trauma exposure and subsequent PTSD symptomatology among war-exposed individuals was moderated by ER flexibility. Specifically, individuals with low ER flexibility exhibited a significant correlation between trauma-exposure and PTSD symptoms, while those with high ER flexibility did not exhibit a dose-response relationship between trauma exposure and subsequent PTSD symptoms. Our findings extend this prior correlational research to demonstrate that trauma-affected participants with probable PTSD who adopted an ER flexible approach when confronted with trauma-salient stressors exhibited small yet significant improvements in emotional responding. This highlights that experimentally inducing higher ER flexibility in individuals with probable PTSD conferred psychological benefits. Together, these findings may suggest that ER flexibility may be a viable mechanism underpinning PTSD that warrants further investigation.

Research investigating the role of ER flexibility in the development of PTSD may be fruitful in elucidating components of emotion regulation that facilitate recovery. Well-established cognitive and cognitive-behavioural models of PTSD consistently identify deficits in emotion regulation as key maintaining factors (Ehlers & Clark, 2000; Foa et al., 1989). Although investigations of ER flexibility in the context of PTSD are in their infancy, key characteristics of PTSD typify ER flexibility deficits, including a limited ER repertoire (marked by an over-reliance on avoidance-based emotion regulation strategies) and limited contextual sensitivity (marked by overgeneralised threat perception and heightened emotional reactivity) (McLean & Foa, 2017; Tull et al., 2020). It is logical then that the provision of explicit guidance in how to employ a more varied ER repertoire that is sensitive to context may result in improved emotional responding specifically among participants with such deficits. This suggests that ER flexibility may be a useful clinical target in interventions for PTSD. Replication of our study, particularly with larger samples of individuals with clinically diagnosed PTSD, is thus an important next step. Although preliminary, findings from this study suggest that ER flexibility training may be a useful adjunct to PTSD treatments. Existing efficacious PTSD treatments, particularly those with ER skills training components such as Skills Training in Affect and Interpersonal Regulation (STAIR; Cloitre et al., 2002) and Dialectical Behaviour Therapy for PTSD (DBT-PTSD; Bohus et al., 2019), may be complemented by including explicit training on *how and when* to use specific ER strategies. In particular, while traditional ER skills training tends to predominantly focus on building a client’s repertoire of effective ER strategies, ER flexibility may be fostered by additionally building skills in perceiving contextual changes (i.e., building an understanding of the different emotional stressors that the client routinely encounters), and building skills in selecting and implementing an appropriate strategy in response to specific stressors (i.e., developing an understanding of which strategies from the client’s personal repertoire best assists with managing symptoms relating to particular stressors). Although tentative, further research may be warranted to explore the potential need for, and effectiveness of, skills training in ER flexibility.

### Several Limitations are Worth Noting

Firstly, it is noteworthy that several valid conceptualisations of ER flexibility exist. The present study was informed by Aldao and colleagues’ (2015) conceptualisation, which predominantly focuses on one’s strategy repertoire and context sensitivity. However, Bonanno and Burton (2013) have conceptualised ER flexibility as the interplay between three key components, namely strategy repertoire, context sensitivity and feedback responsiveness. Feedback responsiveness was not investigated in the present study. Future expansions of this experimental design, that measure a participant’s ability to monitor the efficacy of the instructed ER strategy and adjust if necessary, would be a fruitful avenue for future research. Secondly, given the experimental nature of the study, the instructed ER flexibility paradigm (Specker & Nickerson, 2023) used in the present study employed a narrow operationalisation of ER flexibility. The rationale for this operationalisation was two-fold. First, operationalising ER flexibility using distraction and reappraisal strategies is consistent with prior experimental ER studies and relevant to the presentation of PTSD (Fine et al., 2021; Levy-Gigi et al., 2016; Sheppes, 2020). This is because distraction and reappraisal serve as exemplars of disengagement and engagement strategies, respectively. Thus, keeping this methodological aspect consistent with the broader literature facilitates comparison with existing studies. Second, it was essential to constrain the operationalisation of ER flexibility, both the ER repertoire and the contextual demands of the stimuli, to ensure that we were precisely measuring its impact on subsequent emotional responding. However, a narrow operationalisation of ER limits the generalisability and ecological validity of our findings. Future studies should employ different operationalisations of ER flexibility, such as modifying the contextual demands, or the ER strategies used, to improve the validity of the present findings. Instead of emotional intensity, contextual demands could include one’s personal goals, the perceived controllability of the situation (Troy et al., 2013), or the presence and absence of emotion-evoking cues (Spikol et al., 2024). One potential way to achieve this is to vary the images which trigger emotional responding based on personal relevance to the participant. Furthermore, to improve the ecological validity of the emotional triggers, richer more evocative emotional stimuli such as a video or personally relevant stimuli could be used. Alternatively, different ER strategies may be used instead of distraction and reappraisal, such as suppression and acceptance (Aldao et al., 2010).

Thirdly, there may be limitations in the external validity of our sample. The sample of participants recruited in the present study contained a small percentage of those with probable PTSD. Additionally, our participants were recruited via MTurk. While MTurk samples can recruit reliable and comparable trauma-exposed samples (Engle et al., 2020), we note that our sample largely comprised highly-educated white males. This differs from the demographic characteristics typically seen in the general PTSD population in the US, which tends to comprise a larger proportion of female, non-white, and lower-income individuals (Schein et al., 2021). Accordingly, the present sample may not be generalisable to the broader PTSD population in the US. A potential reason for this discrepancy may be the stringent data validity checks used in the present study, which required participants to have completed a large number of prior MTurk studies (1000 Human Intelligence Tasks [HITs]) to be eligible for the present study. This criterion was included to safeguard a high level of data quality, but may have had the unintended consequence of biasing the sample towards individuals that had more spare time to dedicate to online research. Considering our findings, it may be worthwhile to replicate the current study using purposive sampling to obtain a larger, and potentially more representative, sample of individuals with PTSD. Additionally, while our sample comprised participants with a diversity of traumatic experiences, the specific influences of different types of traumatic events (e.g., transportation accident vs. physical assault) or different levels of exposure (e.g., directly experienced vs. witnessed) on the relationship between instructed ER flexibility, probable PTSD and negative affect were not investigated. Owing to the small sample size of the current study, it was not feasible to conduct such fine-grain analysis. However, such investigations are important to advancing our understanding of when, and for whom, instructed ER flexibility may be most helpful, and thus constitute a worthwhile avenue for future research.

Finally, measurement of key constructs could also be improved. The present study used the PTSD Checklist for the DSM-5 to screen participants with a probable diagnosis of PTSD(PCL-5; American Psychiatric Association, 2013) as it is the gold-standard measure of probable PTSD. However, the PCL-5 is a self-report questionnaire which may limit its response validity. To improve this, the Clinician Administered PTSD Scale for DSM-5 may be a more suitable measure (CAPS-5; Weathers et al., 2018). The CAPS-5 is a widely used structured diagnostic interview that is implemented by trained clinicians to diagnose posttraumatic stress disorder and measure symptom severity (Weathers et al., 2018). Additionally, a single-item measure of negative affect was used to measure the impact of ER flexibility on emotional responding. Although this accords with conventions in the field (Sheppes, 2020), the impact of ER flexibility on emotional responding could be more comprehensively captured by multidimensional methods of assessment. For instance, psychophysiological data such as skin conductance may be collected as an objective, biological marker of emotion dysregulation via autonomic nervous system activity (Fitzgerald et al., 2022). Alternatively, a more comprehensive measure of negative emotion may be used, such as the negative affect scale of the Positive and Negative Affect Schedule (PANAS; Watson et al., 1988). The negative items of the PANAS capture emotions such as anger and guilt (Watson et al., 1988), which are both highly associated with the persistent negative emotional states experienced by those with PTSD (Badour et al., 2017).

Notwithstanding these limitations, the present findings offer preliminary evidence that ER flexibility is a viable mechanism implicated in PTSD. We sought to investigate whether experimentally manipulating ER flexibility would influence emotional responding among trauma-affect participants. Our results demonstrated that trauma-exposed individuals with probable PTSD benefitted from being instructed in using an ER flexible approach, characterised by a wider strategy repertoire and improved context sensitivity. These findings implicate ER flexibility as a potential mechanism underpinning PTSD and illuminate ER flexibility as potentially relevant clinical target in PTSD treatment.

## Electronic Supplementary Material

Below is the link to the electronic supplementary material.

Supplementary Material 1

## Footnotes

1 Reappraisal is an ER strategy that involves disengaging from an emotional stimulus by diverting one’s attention away from the emotional stimulus, to reduce the intensity of the emotional response (Van Dillen & Koole, 2007).

2 Distraction is an ER strategy that involves engaging with an emotional stimulus, and changing the way one thinks about the emotional stimulus, to reduce the intensity of the emotional response (Gross & John, 2003).

## References

[CR1] Aguinis, H., Villamor, I., & Ramani, R. S. (2021). MTurk research: Review and recommendations. *Journal of Management*, *47*(4), 823–837. 10.1177/0149206320969787

[CR2] Aldao, A., Nolen-Hoeksema, S., & Schweizer, S. (2010). Emotion-regulation strategies across psychopathology: A meta-analytic review. *Clinical Psychology Review*, *30*(2), 217–237. 10.1016/j.cpr.2009.11.004 20015584 10.1016/j.cpr.2009.11.004

[CR3] Aldao, A., Sheppes, G., & Gross, J. J. (2015). Emotion regulation flexibility. *Cognitive Therapy and Research*, *39*(3), 263–278. 10.1007/s10608-014-9662-4

[CR4] American Psychiatric Association (2013). *Diagnostic and statistical manual of mental disorders (5th ed.)*. 10.1176/appi.books.9780890425596

[CR51] Ashbaugh, A. R., Houle-Johnson, S., Herbert, C., El-Hage, W., & Brunet, A. (2016). Psychometric validation of the English and French versions of the Posttraumatic Stress Disorder Checklist for DSM-5 (PCL-5). *PLoS ONE*, *11*(10). 10.1371/JOURNAL.PONE.0161645 10.1371/journal.pone.0161645 PMC5056703 27723815

[CR5] Badour, C. L., Resnick, H. S., & Kilpatrick, D. G. (2017). Associations between specific negative emotions and DSM-5 PTSD among a national sample of interpersonal trauma survivors. *Journal of Interpersonal Violence*, *32*(11), 1620. 10.1177/0886260515589930 26088902 10.1177/0886260515589930 PMC4769114

[CR6] Benjamini, Y., & Hochberg, Y. (1995). Controlling the false discovery rate: a practical and powerful approach to multiple testing. *Journal of the Royal statistical society: series B (Methodological)*, *57*(1), 289-300. 10.1186/s40479-019-0099-y

[CR37] Bohus, M., Schmahl, C., Fydrich, T., Steil, R., Müller-Engelmann, M., Herzog, J.,Ludäscher, P., Kleindienst, N., & Priebe, K. (2019). A research programme to evaluate DBT-PTSD, a modular treatment approach for Complex PTSD after childhood abuse. *Borderline personality disorder and emotion dysregulation*, *6*(1), 7. 10.1186/s40479-019-0099-y 10.1186/s40479-019-0099-y PMC6402166 30873283

[CR7] Bonanno, G. A., & Burton, C. L. (2013). Regulatory flexibility: An individual differences perspective on coping and emotion regulation. *Perspectives on Psychological Science*, *8*(6), 591–612. 10.1177/1745691613504116 26173226 10.1177/1745691613504116

[CR52] Bradley, M. M., & Lang, P. J. (2017). *International affective picture system. In Encyclopedia of Personality and Individual Differences*, (pp. 1–4). Springer International Publishing. 10.1007/978-3-319-28099-8_42-1

[CR8] Burton, C. L., & Bonanno, G. A. (2016). Measuring ability to enhance and suppress emotional expression: The flexible regulation of emotional expression (FREE) scale. *Psychological Assessment*, *28*(8), 929–941. 10.1037/PAS0000231 26502200 10.1037/pas0000231

[CR9] Chen, S., & Bonanno, G. A. (2021). Components of emotion regulation flexibility: Linking latent profiles to depressive and anxious symptoms. *Clinical Psychological Science*, *9*(2), 236–251. 10.1177/2167702620956972

[CR39] Chen, M. S., Bi, K., Han, X., Sun, P., & Bonanno, G. A. (2024). Emotion regulation flexibility and momentary affect in two cultures. *Nature Mental Health*, *2*(4), 450–459.

[CR10] Cheng, C. (2001). Assessing coping flexibility in real-life and laboratory settings: A multimethod approach. *Journal of Personality and Social Psychology*, *80*(5), 814–833. 10.1037//0022-3514.80.5.814 10.1037//0022-3514.80.5.814 11374752

[CR11] Cloitre, M., Koenen, K., Cohen, L., & Han, H. (2002). Skills training in affective and interpersonal regulation followed by exposure: A phase-based treatment for PTSD related to childhood abuse. *Journal of Consulting and Clinical Psychology*, *70*(5), 1067–1074. 12362957 10.1037//0022-006x.70.5.1067

[CR12] Ehlers, A., & Clark, D. M. (2000). A cognitive model of posttraumatic stress disorder. *Behaviour Research and Therapy*, *38*(4), 319–345. 10.1016/S0005-7967(99)00123-0 10761279 10.1016/s0005-7967(99)00123-0

[CR40] Ehring, T., Limburg, K., Kunze, A. E., Wittekind, C. E., Werner, G. G., Wolkenstein, L., Guzey, M., & Cludius, B. (2022). (When and how) does basic research in clinical psychology lead to more effective psychological treatment for mental disorders? *Clinical Psychology Review*, *95*, 102163. 10.1016/j.cpr.2022.102163 10.1016/j.cpr.2022.102163 35660924

[CR13] Engle, K., Talbot, M., & Samuelson, K. W. (2020). Is Amazon’s mechanical Turk (MTurk) a comparable recruitment source for trauma studies? *Psychological Trauma: Theory Research Practice and Policy*, *12*(4), 381. 31380674 10.1037/tra0000502

[CR41] English, T., & Eldesouky, L. (2020). Emotion regulation flexibility: Challenges and promise of using ecological momentary assessment. *European Journal of Psychological Assessment*, *36*(3), 456–459. 10.1027/1015-5759/a000581

[CR14] Fine, N. B., Ben-Aharon, N., Armon, D. B., Seligman, Z., Helpman, L., Bloch, M., Hendler, T., & Sheppes, G. (2021). Reduced emotion regulatory selection flexibility in post-traumatic stress disorder: Converging performance-based evidence from two PTSD populations. *Psychological Medicine*. 10.1017/S0033291721004670 37449489 10.1017/S0033291721004670 PMC10244008

[CR15] Fitzgerald, J. M., Timmer-Murillo, S., Sheeran, C., Begg, H., Christoph, M., deRoon-Cassini, T. A., & Larson, C. L. (2022). Psychophysiological predictors of change in emotion dysregulation 6 months after traumatic injury. *International Journal of Psychophysiology*, *173*, 29–37. 10.1016/J.IJPSYCHO.2022.01.005 35007667 10.1016/j.ijpsycho.2022.01.005

[CR16] Foa, E. B., Steketee, G., & Rothbaum, B. O. (1989). Behavioral/cognitive conceptualizations of post-traumatic stress disorder. *Behavior Therapy*, *20*(2), 155–176. 10.1016/S0005-7894(89)80067-X

[CR43] Gamble, K. R., Howard, J. H., & Howard, D. V. (2014). Not just scenery: Viewing nature pictures improves executive attention in older adults. *Experimental Aging Research*, *40*(5), 513. 10.1080/0361073X.2014.956618 10.1080/0361073X.2014.956618 PMC4929355 25321942

[CR44] Gray, M. J., Litz, B. T., Hsu, J. L., & Lombardo, T. W. (2004). Psychometric properties of the Life Events Checklist (LEC-5). *Assessment*, *11*(4), 330–341. 10.1177/1073191104269954 10.1177/1073191104269954 15486169

[CR17] Gross, J. J., & John, O. P. (2003). Individual differences in two emotion regulation processes: Implications for affect, relationships, and well-being. *Journal of Personality and Social Psychology*, *85*(2), 348–362. 10.1037/0022-3514.85.2.348 10.1037/0022-3514.85.2.348 12916575

[CR38] Ioannidis , J. P. A. (2005). Why most published research findings are false. *PLoS Medicine*, *2*(8), e124. 10.1371/journal.pmed.0020124 PMC1182327 16060722

[CR45] Jobson, L., Willoughby, C., Specker, P., Wong, J., Draganidis, A., Lau, W., & Liddell, B. (2022). Investigating the associations between cognitive appraisals, emotion regulation and symptoms of posttraumatic stress disorder among Asian American and European American trauma survivors. *Scientific Reports*, *12*(1). 10.1038/s41598-022-22995-3 10.1038/s41598-022-22995-3 PMC9616820 36307529

[CR18] Kanske, P., Heissler, J., Schönfelder, S., Bongers, A., & Wessa, M. (2011). How to regulate emotion? Neural networks for reappraisal and distraction. *Cerebral Cortex*, *21*(6), 1379–1388. 10.1093/CERCOR/BHQ216 21041200 10.1093/cercor/bhq216

[CR19] Kashdan, T. B., & Rottenberg, J. (2010). Psychological flexibility as a fundamental aspect of health. *Clinical Psychology Review*, *30*(7), 865–878. 10.1016/J.CPR.2010.03.001 21151705 10.1016/j.cpr.2010.03.001 PMC2998793

[CR20] Levy-Gigi, E., Bonanno, G. A., Shapiro, A. R., Richter-Levin, G., Kéri, S., & Sheppes, G. (2016). Emotion regulatory flexibility sheds light on the elusive relationship between repeated traumatic exposure and posttraumatic stress disorder symptoms. *Clinical Psychological Science*, *4*(1), 28–39. 10.1177/2167702615577783

[CR46] McDonald, S. D., & Calhoun, P. S. (2010). The diagnostic accuracy of the PTSD Checklist: A critical review. *Clinical Psychology Review*, *30*(8), 976–987. 10.1016/j.cpr.2010.06.012 10.1016/j.cpr.2010.06.012 20705376

[CR21] McLean, C. P., & Foa, E. B. (2017). Emotions and emotion regulation in posttraumatic stress disorder. *Current Opinion in Psychology*, *14*, 72–77. 10.1016/j.copsyc.2016.10.006 28813323 10.1016/j.copsyc.2016.10.006

[CR22] McRae, K., Hughes, B., Chopra, S., Gabrieli, J. D. E., Gross, J. J., & Ochsner, K. N. (2010). The neural bases of distraction and reappraisal. *Journal of Cognitive Neuroscience*, *22*(2), 248. 10.1162/JOCN.2009.21243 19400679 10.1162/jocn.2009.21243 PMC4136451

[CR23] O’Brien, H., Kalokerinos, E. K., Felmingham, K., Lau, W., & O’Donnell, M. (2023). Emotion regulation strategy use in PTSD: A daily life study. *Journal of Affective Disorders*, *338*, 365–372. 10.1016/J.JAD.2023.06.023 37302510 10.1016/j.jad.2023.06.023

[CR24] Perneger, T. V. (1998). What’s wrong with Bonferroni adjustments. *BMJ (Clinical Research Ed)*, *316*(7139), 1236–1238. 9553006 10.1136/bmj.316.7139.1236 PMC1112991

[CR25] Schein, J., Houle, C., Urganus, A., Cloutier, M., Patterson-Lomba, O., Wang, Y., King, S., Levinson, W., Guérin, A., Lefebvre, P., & Davis, L. L. (2021). Prevalence of post-traumatic stress disorder in the United States: A systematic literature review. *Current Medical Research and Opinion*, *37*(12), 2151–2161. 10.1080/03007995.2021.1978417 34498953 10.1080/03007995.2021.1978417

[CR26] Sheppes, G. (2020). Transcending the good & bad and here & now in emotion regulation: Costs and benefits of strategies across regulatory stages. In *Advances in Experimental Social Psychology* (Vol. 61, pp. 185–236). Academic Press Inc. 10.1016/bs.aesp.2019.09.003

[CR27] Sheppes, G., & Meiran, N. (2008). Divergent cognitive costs for online forms of reappraisal and distraction. *Emotion*, *8*(6), 870–874. 10.1037/A0013711 19102598 10.1037/a0013711

[CR28] Sheppes, G., Scheibe, S., Suri, G., & Gross, J. J. (2011). Emotion-regulation choice. *Psychological Science*, *22*(11), 1391–1396. 10.1177/0956797611418350 21960251 10.1177/0956797611418350

[CR50] Sheppes, G., Scheibe, S., Suri, G., Radu, P., Blechert, J., & Gross, J. J. (2014). Emotion regulation choice: A conceptual framework and supporting evidence. *Journal of Experimental Psychology: General*, *143*(1), 163–181. 10.1037/a0030831 10.1037/a0030831 23163767

[CR29] Specker, P., & Nickerson, A. (2023). Investigating the effectiveness of instructing emotion regulation flexibility to individuals with low and high anxiety. *Anxiety Stress and Coping*, 1–14. 10.1080/10615806.2023.2205641 10.1080/10615806.2023.2205641 37120826

[CR30] Specker, P., Sheppes, G., & Nickerson, A. (2023). Does Emotion Regulation Flexibility Work? Investigating the Effectiveness of Regulatory Selection Flexibility in Managing Negative Affect. *Social Psychological and Personality Science*, *15*(5), 561–;569.

[CR31] Spikol, E., McGlinchey, E., Robinson, M., & Armour, C. (2024). Flexible emotional regulation typology: Associations with PTSD symptomology and trait resilience. *BMC Psychology*, *12*(1), 1–10. 10.1186/S40359-024-01573-4/TABLES/5 38365706 10.1186/s40359-024-01573-4 PMC10874029

[CR32] Tull, M. T., Vidaña, A. G., & Betts, J. E. (2020). Emotion regulation difficulties in PTSD. In *Emotion in Posttraumatic Stress Disorder* (pp. 295–310). Elsevier. 10.1016/B978-0-12-816022-0.00010-7

[CR33] Van Dillen, L. F., & Koole, S. L. (2007). Clearing the mind: A working memory model of distraction from negative mood. *Emotion*, *7*(4), 715–723. 10.1037/1528-3542.7.4.715 18039038 10.1037/1528-3542.7.4.715

[CR48] Verhey, R., Chibanda, D., Gibson, L., Brakarsh, J., & Seedat, S. (2018). Validation of the posttraumatic stress disorder checklist in a primary care population with high HIV prevalence in Zimbabwe. *BMC Psychiatry*, *18*(1), 1–8. 10.1186/S12888-018-1688-9/FIGURES/1 10.1186/s12888-018-1688-9 PMC5913864 29685117

[CR34] van Stolk-Cooke, K., Brown, A., Maheux, A., Parent, J., Forehand, R., & Price, M. (2018). Crowdsourcing trauma: Psychopathology in a trauma-exposed sample recruited via Mechanical Turk. *Journal of Traumatic Stress*, *31*(4), 549–557. 10.1002/jts.22303 30025175 10.1002/jts.22303 PMC6107385

[CR35] Watson, D., Clark, L. A., & Tellegen, A. (1988). Development and validation of brief measures of positive and negative affect: The PANAS scales. *Journal of Personality and Social Psychology*, *54*(6), 1063–1070. 10.1037/0022-3514.54.6.1063 3397865 10.1037//0022-3514.54.6.1063

[CR36] Weathers, F. W., Bovin, M. J., Lee, D. J., Sloan, D. M., Schnurr, P. P., Kaloupek, D. G., Keane, T. M., & Marx, B. P. (2018). The clinician-administered PTSD scale for DSM–5 (CAPS-5): Development and initial psychometric evaluation in military veterans. *Psychological Assessment*, *30*(3), 383. 10.1037/PAS0000486 28493729 10.1037/pas0000486 PMC5805662

[CR49] Weathers, F. W., Blake, D. D., Kaloupek, D. G., Marx, B. P., & Keane (2013). *Life Events Checklist for DSM-5 (LEC-5)*. https://www.ptsd.va.gov/professional/assessment/temeasures/life_events_checklist.asp

