# Supplementary material for: An Experimental Investigation of the Relationship Between Emotion Regulation Flexibility, Negative Affect and Posttraumatic Stress Disorder
Source: Cognit Ther Res. 2024 Oct 4;49(2):249–61. doi: 10.1007/s10608-024-10536-3 (PMC11928379; doi:10.1007/s10608-024-10536-3)
Supplement: Supplementary file 1 — Supplementary Material 1 [file 10608_2024_10536_MOESM1_ESM.pdf]

An Experimental Investigation of the Relationship between Emotion Regulation Flexibility, Negative Affect and Posttraumatic Stress Disorder.

ONLINE SUPPLEMENTAL MATERIALS

***Supplement A*:** Response validity indicators

***Supplement B*:** Study instructions and materials

***Supplement C*:** Frequencies of exposure to potentially traumatic events

***Supplement D*:** Pairwise comparisons

Supplement A: Response validity indicators and participant exclusions

The present study implemented several response validity indicators to preserve a high level of data quality. The present study was conducted online via the crowd sourcing platform, MTurk. Thus, we included response validity indicators in line with current recommendations to identify and exclude suspicious (e.g., bots) or inattentive responders (Chmielewski & Kucker, 2020).

Object Check

Despite preventative measures such as reCAPTCHA (a digital test used to distinguish humans from robots) and ballot box stuffing (a setting that prevents responders from completing the survey more than once), all online survey platforms are susceptible to “bots” (Griffin et al., 2022). “Bots” are computer software designed to perform automated tasks like locating and completing high-paying surveys (Teitcher et al., 2015). Since “bots” cannot bypass qualitative items (Griffin et al., 2022), we included an item asking participants to describe an object near them in a few sentences. Responses were screened and flagged if they were (1) repetitive, for example, repetition of identical phrases across multiple items, (2) ungrammatical or nonsensical, or (3) suspicious, for example, plagiarised responses from the internet.

Conscientious Responder Item

To ensure participants attended to the instructions provided, a single item from the Conscientious Responder Scale (Marjanovic et al., 2014) was used, which asked participants to select the answer option “*Quite a bit*” in a multiple-choice question. Respondents who selected any answer options other than “*Quite a bit*” are classified as inattentive responders and excluded from the analysis. In the present study, no participants failed this item.

Image Check

Participants were asked to "Describe the following image", which was a bunch of bananas. This item was a variation of a reCAPTCHA item included to further screen out "bots" that cannot identify items in pictures (Griffin et al., 2022). As in the object check, responses were screened and flagged if they were (1) repetitive, for example, repetition of identical phrases across multiple items, (2) ungrammatical or nonsensical, or (3) suspicious, for example, plagiarised response from the internet.

Supplement B: Study instructions and materials

Distraction Instructions

One of the strategies that you may be asked to use is called: Distraction. When distracting, we want you to try your best to focus all your attention and all of your thoughts on something emotionally neutral. We will now show you some examples of how to use distraction. Please make sure your sound is TURNED ON before clicking the arrow for the next page.

Please click the play button and view this short video. (Please make sure your sound is turned on before playing the video.) The next arrow will appear once you have watched the video. Link to video: https://www.youtube.com/watch?v=Z7wbGAxJyX0

Reappraisal Instructions

One of the strategies that you may be asked to use is called: Reappraisal. When reappraising, we want you to focus on the image, but try your best to change the way you feel by thinking about the meaning of the image in a different way. We will now show you some examples of how to use reappraisal. Please make sure your sound is TURNED ON before clicking the arrow for the next page.

Please click the play button and view this short video. (Please make sure your sound is turned on before playing the video.) The next arrow will appear once you have watched the video. Link to video: https://www.youtube.com/watch?v=r4yGx4Y2Kkk

Supplement C: Frequencies of exposure to potentially traumatic events

Potentially Traumatic Event	Total endorsed (i.e., experienced, witnessed or part of job)		*Experienced*	*Witnessed*	*Part of job*
	N	%	*N (%)*	*N (%)*	*N (%)*
Transportation Accident	114	77	*87 (58.8)*	*62 (41.9)*	*1 (0.7)*
Natural Disaster	84	56.8	*70 (47.3)*	*40 (27)*	*3 (2.0)*
Physical Assault	72	48.6	*53 (35.8)*	*36 (24.3)*	*3 (2.0)*
Life-threatening Illness or Injury	64	43.2	*20 (13.5)*	*50 (33.8)*	*1 (0.7)*
Other Unwanted or Uncomfortable Sexual Experience	54	36.5	*49 (33.1)*	*7 (4.7)*	*2 (1.4)*
Fire or Explosion	53	35.8	*20 (13.5)*	*39 (26.4)*	*1 (0.7)*
Serious Accident at Work, Home, or During Recreational Activity	38	25.7	*18 (12.2)*	*24 (16.2)*	*3 (2.0)*
Sexual Assault	29	19.6	*25 (16.9)*	*5 (3.4)*	*1 (0.7)*
Assault with a Weapon	28	18.9	*15 (10.1)*	*20 (13.5)*	*2 (1.4)*
Other Stressful Event/Experience	27	18.2	*24 (16.2)*	*8 (5.4)*	-
Severe Human Suffering	21	14.2	*5 (3.4)*	*19 (12.8)*	*1 (0.7)*
Sudden Accidental Death	20	13.5	*N/A*	*19 (12.8)*	*2 (1.4)*
Sudden Violent Death	17	11.5	*N/A*	*15 (10.1)*	*2 (1.4)*
Exposure to Toxic Substance	11	7.4	*8 (5.4)*	*1 (0.7)*	*5 (3.4)*
Serious Injury, Harm, or Death You Caused	8	5.4	*2 (1.4)*	*5 (3.4)*	*1 (0.7)*
Combat or Exposure to Warzone	6	4.1	*2 (1.4)*	*2 (1.4)*	*2 (1.4)*
Captivity	3	2	*3 (2)*	*1 (0.7)*	-

Note. N = 148. The frequencies of potentially traumatic events (PTE) listed on the Life Events Checklist for DSM-5 (LEC-5). The sum of PTE exceeds 148 because participants could endorse multiple potentially traumatic events on the LEC-5.

Data and syntax files available from:
https://osf.io/y5kvs/?view_only=c68d860aecb84ac3b4d300a361963783

Supplement D: Pairwise comparisons

PTSD group	Condition 1	Condition 2	Mean Difference	SD	Sig	95% CI Lower Bound	95% CI Upper Bound	Cohen's d
Probable PTSD	ER Flexible	Context Insensitive	-1.575	.708	**.028**	-2.974	-.175	0.14
		Inflexible Distraction	-1.929	.681	**.005**	-3.275	-.583	0.23
		Inflexible Reappraisal	-2.105	1.050	**.047**	-4.182	-.028	0.14
	Context Insensitive	ER Flexible	1.575	.708	**.028**	.175	2.974	0.14
		Inflexible Distraction	-.355	.750	.637	-1.837	1.127	0.19
		Inflexible Reappraisal	-.530	1.097	.629	-2.699	1.638	0.13
	Inflexible Distraction	ER Flexible	1.929	.681	**.005**	.583	3.275	0.23
		Context Insensitive	.355	.750	.637	-1.127	1.837	0.19
		Inflexible Reappraisal	-.176	1.077	.871	-2.306	1.954	0.14
	Inflexible Reappraisal	ER Flexible	2.105	1.050	**.047**	.028	4.182	0.14
		Context Insensitive	.530	1.097	.629	-1.638	2.699	0.13
		Inflexible Distraction	.176	1.077	.871	-1.954	2.306	0.14
No Probable PTSD	ER Flexible	Context Insensitive	.199	.354	.575	-.500	.898	0.16
		Inflexible Distraction	.042	.355	.905	-.660	.744	0.20
		Inflexible Reappraisal	.664	.337	.051	-.003	1.332	0.18
	Context Insensitive	ER Flexible	-.199	.354	.575	-.898	.500	0.16
		Inflexible Distraction	-.157	.351	.657	-.851	.538	0.22
		Inflexible Reappraisal	.465	.337	.170	-.202	1.133	0.20
	Inflexible Distraction	ER Flexible	-.042	.355	.905	-.744	.660	0.20
		Context Insensitive	.157	.351	.657	-.538	.851	0.22
		Inflexible Reappraisal	.622	.338	.068	-.046	1.290	0.21
	Inflexible Reappraisal	ER Flexible	-.664	.337	.051	-1.332	.003	0.18
		Context Insensitive	-.465	.337	.170	-1.133	.202	0.20
		Inflexible Distraction	-6.22	.338	.068	-1.290	.046	0.21

Note. Pairwise comparisons of mean negative affect across experimental conditions and PTSD groups. Data and syntax files available from: https://osf.io/y5kvs/?view_only=c68d860aecb84ac3b4d300a361963783
